# Warming-induced permafrost thaw exacerbates tundra soil carbon decomposition mediated by microbial community

**DOI:** 10.1186/s40168-019-0778-3

**Published:** 2020-01-17

**Authors:** Jiajie Feng, Cong Wang, Jiesi Lei, Yunfeng Yang, Qingyun Yan, Xishu Zhou, Xuanyu Tao, Daliang Ning, Mengting M. Yuan, Yujia Qin, Zhou J. Shi, Xue Guo, Zhili He, Joy D. Van Nostrand, Liyou Wu, Rosvel G. Bracho-Garillo, C. Ryan Penton, James R. Cole, Konstantinos T. Konstantinidis, Yiqi Luo, Edward A. G. Schuur, James M. Tiedje, Jizhong Zhou

**Affiliations:** 1 Institute for Environmental Genomics, Department of Microbiology and Plant Biology, University of Oklahoma, Norman, OK 73019 USA; 2 State Key Joint Laboratory of Environment Simulation and Pollution Control, School of Environment, Tsinghua University, Beijing, 100084 China; 3 Environmental Microbiomics Research Center, School of Environmental Science and Engineering, Sun Yat-Sen University, Guangzhou, 510006 China; 4 Guangdong Provincial Key Laboratory of Environmental Pollution Control and Remediation Technology, School of Environmental Science and Engineering, Sun Yat-Sen University, Guangzhou, 510006 China; 5 School of Minerals Processing and Bioengineering, Central South University, Changsha, 410083 China; 6 School of Forest Resources and Conservation, University of Florida, Gainesville, FL 32611 USA; 7 Center for Fundamental and Applied Microbiomics, Arizona State University, Mesa, AZ 85212 USA; 8 College of Integrative Sciences and Arts, Faculty of Science and Mathematics, Arizona State University, Mesa, Arizona 85212 USA; 9 Center for Microbial Ecology, Michigan State University, East Lansing, MI 48824 USA; 10 School of Civil and Environmental Engineering, School of Biology, and Center for Bioinformatics and Computational Genomics, Georgia Institute of Technology, Atlanta, GA 30332 USA; 11 Center for Ecosystem Science and Society, Northern Arizona University, Flagstaff, AZ 86011 USA; 12 DOE Great Lakes Bioenergy Research Center, Michigan State University, East Lansing, MI 48824 USA; 13 Earth and Environmental Sciences, Lawrence Berkeley National Laboratory, Berkeley, CA 94720 USA

## Abstract

**Background:**

It is well-known that global warming has effects on high-latitude tundra underlain with permafrost. This leads to a severe concern that decomposition of soil organic carbon (SOC) previously stored in this region, which accounts for about 50% of the world’s SOC storage, will cause positive feedback that accelerates climate warming. We have previously shown that short-term warming (1.5 years) stimulates rapid, microbe-mediated decomposition of tundra soil carbon without affecting the composition of the soil microbial community (based on the depth of 42684 sequence reads of 16S rRNA gene amplicons per 3 g of soil sample).

**Results:**

We show that longer-term (5 years) experimental winter warming at the same site altered microbial communities (*p* < 0.040). Thaw depth correlated the strongest with community assembly and interaction networks, implying that warming-accelerated tundra thaw fundamentally restructured the microbial communities. Both carbon decomposition and methanogenesis genes increased in relative abundance under warming, and their functional structures strongly correlated (*R*^2^ > 0.725, *p* < 0.001) with ecosystem respiration or CH_4_ flux.

**Conclusions:**

Our results demonstrate that microbial responses associated with carbon cycling could lead to positive feedbacks that accelerate SOC decomposition in tundra regions, which is alarming because SOC loss is unlikely to subside owing to changes in microbial community composition.

Video Abstract

## Background

High-latitude permafrost-underlain tundra ecosystems have been a hotspot for climate change research, owing to their substantial carbon (C) pool and high vulnerability to climate warming [1–4]. Old C from plant and animal remnants has been sequestered in permafrost regions for thousands of years under frozen soil conditions [5]. Although accounting for only 15% of the total global land mass, the northern hemisphere permafrost regions at a depth of 0–3 m contain 1 672 Pg C, roughly half of the global soil C pool [2, 6]. Since permafrost regions have the potential to release a large amount of previously stored soil C to the atmosphere in a warmer world [3, 4], it is a significant variable that affects the future trajectory of climate change [7].

Over the past 30 years, annual average temperatures in high latitude regions have increased by 0.6 °C per decade, twice as fast as the global average [8], resulting in the substantial thaw of permafrost soils. It has been estimated that climate warming will cause a reduction of 30–70% of the total permafrost soils by the end of the twenty-first century [9]. As a consequence, previously protected soil C becomes available for microbial decomposition [2]. A number of studies have shown that tundra soil C is highly vulnerable and responds rapidly to the warming-induced thaw of permafrost soils [2, 4, 10]. Although the increase in soil C input by higher plant productivity across the tundra regions could partially offset soil C loss [11–13], there remains a lack of a mechanistic understanding of microbial responses to climate warming, which makes it challenging to assess the future C balance.

Only a few studies of permafrost ecosystems have examined microbial responses to climate warming [4, 7, 14]. For example, a substantial fraction of permafrost soil C was available for microbe-mediated decomposition during a lab incubation simulating warming [15]. Consistently, a field study in a permafrost-based tundra (the same site as this study) revealed that microbial community functional potential was highly sensitive to a 1.5-year experimental warming, despite the taxonomic composition remaining unaltered [4]. As a result, soil C was more vulnerable to microbial decomposition. However, it remains unclear whether microbial responses to short-term warming persist in the longer term.

Since a 1.5-year warming altered the microbial functional structure but not the taxonomic composition of soil microbial communities in permafrost-based tundra [4], our central hypothesis was that 5 years’ warming could induce changes in plant productivity, soil microclimate, and soil microbial community structure. We expected three mutually exclusive outcomes after longer-term warming: (i) similar to that observed after the 1.5-year warming period, the microbial functional structure would be altered, while the taxonomic composition would remain similar to that of the control group (resistance); (ii) the microbial communities that are acclimated to experimental warming would manifest a functional structure and taxonomic composition that approximates that of the control group (resilience); or (iii) microbial communities would continue to evolve into new states and both functional structure and taxonomic composition would be altered by warming (sensitivity).

To test our hypothesis, we examined soil microbial communities subjected to a 5-year winter warming treatment at the Carbon in Permafrost Experimental Heating Research (CiPEHR) site located in Alaska, USA. This site has been extensively used to analyze the effects of climate warming on plants, soil nitrogen (N) availability, and soil microbial communities [4, 16–18]. A winter warming treatment was carried out by snow fences (1.5 m tall and 8 m long), which warmed the soil by maintaining thick snow layers as heat insulators. We investigated both the taxonomic composition and functional structure of microbial communities under warming, in addition to potential drivers and ecological consequences of community changes. Specifically, the taxonomic composition of microbial communities was investigated by amplicon sequencing of 16S rRNA genes for bacterial/archaeal community and internal transcribed spacer (ITS) region for the fungal community. The microbial functional structure was examined by a functional microarray named GeoChip 5.0M [19].

## Results

### Edaphic factors, plant productivity, and ecosystem C fluxes

As shown in Additional file 1: Table S1, the average winter soil temperature increased by 0.63 °C (*p* = 0.037) under warming and the maximum thaw depth increased by 11.37 cm (*p* = 0.006), much more substantial than the 4.78 cm increase after the 1.5-year warming [4]. Aboveground plant biomass at the end of the growth season increased by 25.2% (*p* = 0.049) under warming, similar to other observations in tundra regions [11–13]. Ecosystem respiration increased by 72.8% (*p* < 0.001) under warming, and CH_4_ flux increased by 218.8% (*p* = 0.004).

### Microbial community composition

We examined the taxonomic composition of microbial communities via high-throughput amplicon sequencing of bacterial and archaeal 16S rRNA genes and the fungal internal transcribed spacer (ITS) region. After resampling at 34 673 reads per sample, 5 117 OTUs were generated by 16S rRNA gene amplicon sequencing. Almost all of the OTUs (99.86%) and relative abundance (99.88%) belonged to bacteria, with 2 740 OTUs mapping to 214 known genera. *Proteobacteria* was the most abundant phylum (31.00% in relative abundance), followed by *Acidobacteria* (30.61%), *Actinobacteria* (12.08%), and *Verrucomicrobia* (8.34%) (Additional file 1: Figure S1a). Among *Proteobacteria*, the relative abundance of *Alphaproteobacteria* was 13.86% and that of *Gammaproteobacteria* was 7.74%. For fungi, 1 465 OTUs were generated by ITS amplicon sequencing after resampling at 19 242 reads per sample. *Leotiomycetes* were the most abundant class (47.35% in relative abundance), followed by *Eurotiomycetes* (18.85%), unidentified *Ascomycota* (16.06%), and *Agaricomycetes* (10.05%) (Additional file 1: Figure S1b).

**Fig. 1:**
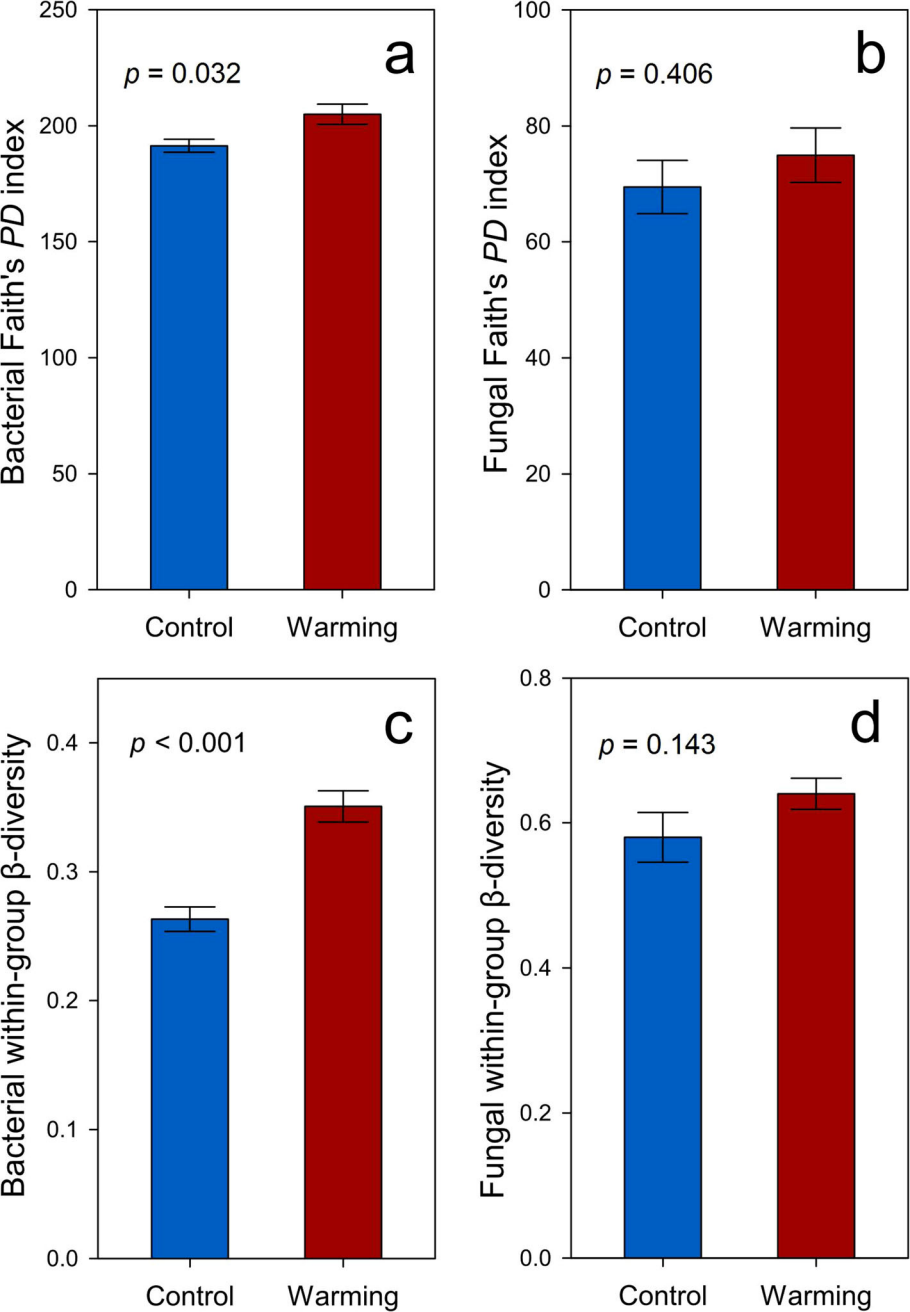
Diversity indices of bacterial/fungal communities, including **a** bacterial Faith’s *PD* index (phylogenetic α-diversity index), **b** fungal Faith’s *PD* index, **c** bacterial within-group β-diversity (Bray-Curtis distance), and **d** fungal within-group β-diversity (Bray-Curtis distance). Statistical significances were determined by permutation *t* tests. Error bars represent standard error of the mean for *n* = 6 biological replicates

Warming increased the phylogenetic α-diversity of the bacterial communities (Faith’s *PD*, *p* = 0.032, Fig. 1a) but not the fungal communities, probably due to high fungal variance among a limited number of biological replicates (*p* = 0.406, Fig. 1b). Bacterial within-group β-diversity, i.e., the difference within biological replicates, was also increased in warmed samples (*p* < 0.001, Fig. 1c), indicating that warming led to more divergent bacterial communities. In contrast, fungal within-group β-diversity remained unchanged (*p* = 0.143, Fig. 1d). All of the nonparametric multivariate statistical tests of dissimilarity (MRPP, ANOSIM, and Adonis) showed that warming altered the composition of the bacterial communities but not the fungal communities (*p* < 0.040, Table 1).

**Table 1:** Dissimilarity tests of warming effects on microbial taxonomic composition revealed by 16S rRNA gene and ITS sequencing, and functional structure revealed by GeoChip

Dataset	MRPPa		ANOSIM		Adonis	
	delta	*p*	r	*p*	r2	*p*
16S rRNA gene	1338.991	**0.040b**	0.152	**0.028**	0.162	**0.015**
ITS	0.610	0.741	0.067	0.722	0.070	0.738
GeoChip	0.001	**0.012**	0.296	**0.012**	0.166	**0.009**

^a^Three permutation tests were performed, including the multiple response permutation procedure (MRPP), analysis of similarity (ANOSIM), and permutational multivariate analysis of variance (Adonis). Bray-cutis distance was used in the permutation tests

^b^Bold values indicate *p* < 0.050

### Microbial correlation networks

All bacterial and fungal networks generated from control or warmed samples showed topological properties of small-world, scale-free, and modularity, and were significantly different from randomly generated networks (Additional file 1: Table S2). The average connectivity of the bacterial network in warmed samples was higher (*p* < 0.001), but the average geodesic distance was lower (*p* < 0.001) than those in the control samples, suggesting that nodes were more connected in warmed samples. In contrast, the average connectivity and the average geodesic distance of fungal networks were reduced by warming (*p* < 0.001), owing to increased network modularity (Additional file 1: Table S2).

To explore the relationship between network topology and environmental factors, we included environmental factors as nodes in the networks. Thaw depth had the highest node connectivity in the bacterial network of warmed samples (Additional file 1: Figure S2a), while water table depth had the highest node connectivity in the bacterial network of control samples (Additional file 1: Figure S2b). In contrast, thaw depth, bulk density and soil N had the highest node connectivity in the fungal network of warmed samples (Additional file 1: Figure S2c), while bulk density and soil N showed the highest node connectivity in the fungal network of control samples (Additional file 1: Figure S2d).

### Microbial community functional structure

**Fig. 2:**
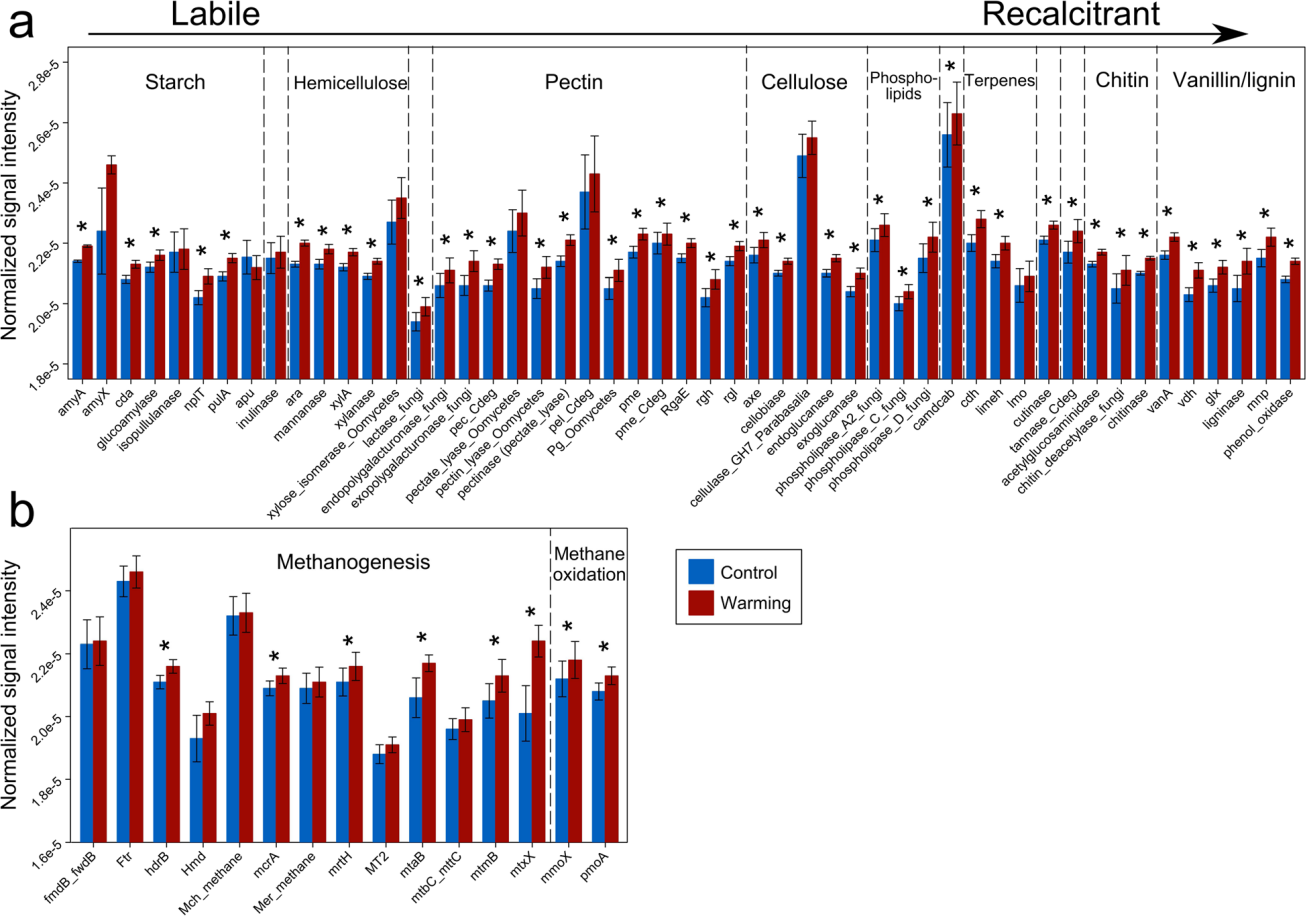
Normalized signal intensities of representative genes involved in **a** C decomposition and **b** methane cycling, as revealed by GeoChip 5.0 analysis. Blue bars represent the average normalized signal intensity of probes of each gene of control samples, and red bars represent warmed samples. Error bars represent standard error of the mean for *n* = 6 biological replicates. The differences of the functional gene relative abundance between warming and control samples were tested using ANOVA, indicated by * when *p* < 0.050

A total of 38 484 probes on the GeoChip showed positive signals. All of the nonparametric multivariate statistical tests of dissimilarity (MRPP, ANOSIM, and Adonis) showed that the overall functional structure of soil microbial communities was altered by warming (*p* < 0.012, Table 1), and positively correlated with bacterial and fungal community composition (*p* < 0.015, Additional file 1: Figure S3). The relative abundance of genes associated to C, N, phosphorus (P), and sulfur (S) cycling was increased by warming (Fig. 2 and Additional file 1: Figure S4). In contrast, only nine functional genes, which mainly belong to functions related to virulence and virus, were significantly (*p* < 0.05) decreased in relative abundance.

#### C cycling

We detected 50 genes associated with decomposition of labile or recalcitrant C. Among them, 42 genes exhibited higher relative abundance in warmed samples than control samples (*p* < 0.038, Fig. 2a), including *amyA* encoding amylase, *xylA* encoding xylose isomerase, exoglucanase, cellobiase, pectate lyase, phenol oxidase, *vdh* encoding vanillin dehydrogenase, and ligninase.

A total of 13 methanogenesis genes were detected (Fig. 2b). Among them, *mcrA* encoding methyl coenzyme M reductase, *mrtH* encoding tetrahydromethanopterin S-methyltransferase, *mtaB* encoding methanol-cobalamin methyltransferase, *mtmB* encoding monomethylamine methyltransferase, *mtxX* encoding methyltransferase, and *hdrB* encoding CoB/CoM heterodisulfide reductase exhibited higher relative abundance in warmed samples (*p* < 0.007), suggesting a higher functional potential of methanogenesis. In addition, both methane oxidation genes, which are *mmoX* encoding soluble methane monooxygenase and *pmoA* encoding particulate methane monooxygenase, exhibited higher relative abundance in warmed samples (*p* < 0.001, Fig. 2b).

**Fig. 3:**
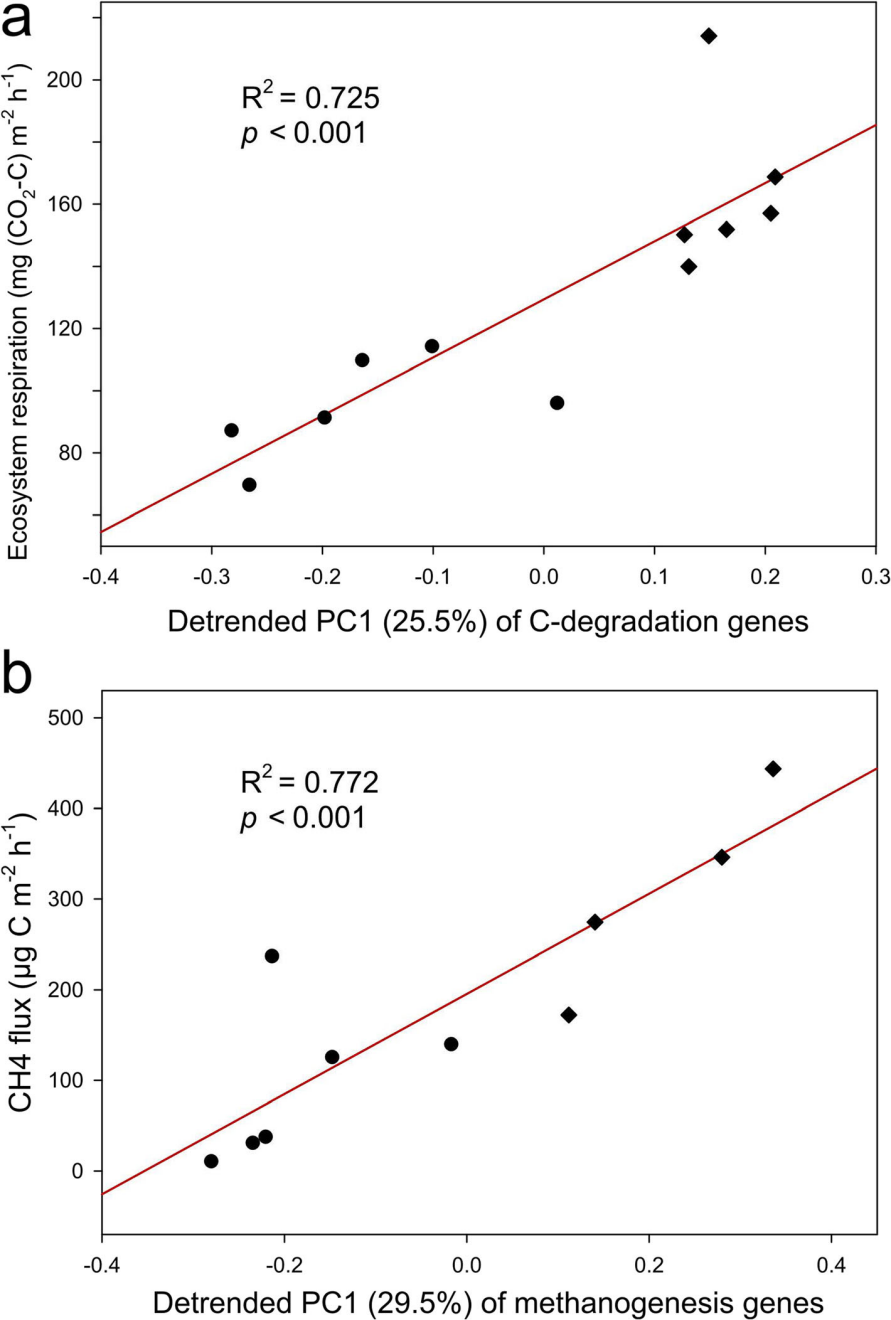
Linear regressions between **a** in situ ecosystem respiration and the first detrended principle component (PC1) of C decomposition genes, and **b** in situ methane flux and PC1 of methanogenesis genes. Each point represents a biological replicate of warming (diamonds) or control (circles) samples

Higher functional capacities of microbial C degradation and methanogenesis in warmed samples could lead to in situ C loss. Accordingly, we detected strong correlations between functional structure of C decomposition genes and in situ ecosystem respiration (*R*^2^ = 0.725, *p* < 0.001, Fig. 3a), and between the functional structure of methanogenesis genes and in situ CH_4_ flux (*R*^2^ = 0.772, *p* < 0.001, Fig. 3b).

#### N cycling

As a limiting nutrient in tundra ecosystems, N plays an essential role in ecosystem productivity. All the detected genes associated with N cycling exhibited higher relative abundance in warmed samples (*p* < 0.025, Additional file 1: Figure S4a), suggesting that warming enhanced microbial functional capacity for N cycling. These genes included the N fixation gene (*nifH* encoding nitrogenase reductase), nitrification gene (*hao* encoding hydroxylamine oxidoreductase), denitrification genes (e.g., *narG* encoding nitrate reductase), dissimilatory nitrate reduction genes (e.g., *napA* encoding periplasmic nitrate reductase), assimilatory nitrate reduction genes (e.g., *nasA* encoding assimilatory nitrate reductase), N mineralization gene (*ureC* encoding urease), and ammonia assimilation gene (*gdh* encoding glutamate dehydrogenase).

#### P and S cycling

P deficiency is common in global soil ecosystems. We found that P cycling genes including phytase and *ppx* encoding exopolyphosphatase (*ppx*) were in higher relative abundance in the warmed samples (*p* < 0.001, Additional file 1: Figure S4b), suggesting that warming could potentially increase microbial functional capacity of P cycling. Similarly, 27 genes associated with S cycling were detected, of which 21 showed higher relative abundance in warmed samples (*p* < 0.027, Additional file 1: Figure S4c). These genes included *dsrA*/*B*-encoding dissimilatory sulfite reductase, *SiR*- and *cysI*/*J*-encoding sulfate reductase, and *soxY*-encoding sulfur oxidation protein.

### Microbial community assembly mechanisms and the importance of thaw depth

To assess the importance of deterministic and stochastic processes in shaping soil community composition, stochastic ratios were calculated. Stochastic processes of bacterial communities were reduced by warming from 91.5 to 65.9% (*p* < 0.001, Additional file 1: Figure S5a), suggesting that environmental filtering was elicited by warming. Similarly, stochastic ratios of fungal communities were reduced by warming (*p* = 0.036, Additional file 1: Figure S5b).

**Fig. 4:**
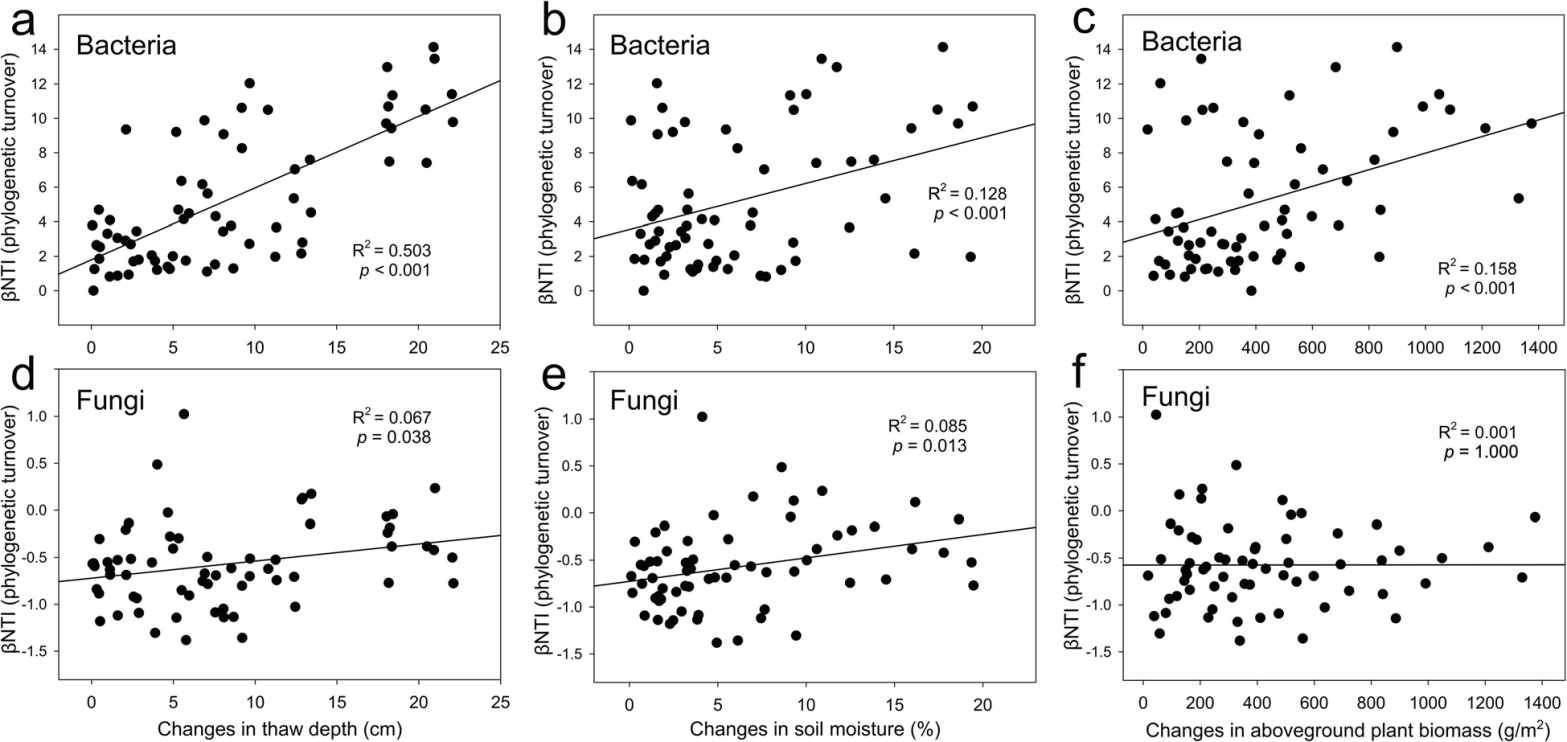
Linear regressions between pairwise microbial community phylogenetic turnovers (Beta Nearest Taxon Index, βNTI) and pairwise differences of plant and soil factors. Phylogenetic turnover metrics are related to changes in **a** soil thaw depth, **b** soil moisture and **c** aboveground plant biomass for bacterial communities, and changes in **d** soil thaw depth, **e** soil moisture and **f** aboveground plant biomass for fungal communities. The 66 points in each sub-figure represent the 66 pairwise differences generated from the 6 warmed samples and 6 control samples

To identify environmental factors that may have a strong effect on the microbial communities, we performed correlation tests between the beta-nearest taxon index (βNTI, also known as phylogenetic β-diversity) [20] and pairwise differences in all of 14 environmental factors. Bacterial βNTI correlated with the thaw depth (*R*^2^ = 0.503, *p* < 0.001, Fig. 4a), and to a lesser extent with soil moisture (*R*^2^ = 0.128, *p* < 0.001, Fig. 4b) and aboveground plant biomass (*R*^2^ = 0.158, *p* < 0.001, Fig. 4c). Fungal βNTI had weaker correlations with those factors than bacterial βNTI, but correlated with thaw depth (*R*^2^ = 0.067, *p* = 0.038, Fig. 4d) and soil moisture (*R*^2^ = 0.085, *p* = 0.013, Fig. 4e) while not with aboveground plant biomass (*R*^2^ = 0.001, *p* = 1.000, Fig. 4f).

We performed CCA to verify the importance of the thaw depth in microbial community assembly. The bacterial community composition correlated with thaw depth, aboveground plant biomass, soil moisture, and winter soil temperature, with soil moisture and aboveground plant biomass being the most important variables (*p* = 0.007, Additional file 1: Figure S6a). Similarly, thaw depth, aboveground plant biomass, soil moisture, winter soil temperature and soil C/N ratio correlated with the fungal community composition (*p* = 0.012, Additional file 1: Figure S6b) and with the microbial functional structure (*p* < 0.001, Additional file 1: Figure S6c).

## Discussion

Given the tremendous amount of soil C stored within permafrost regions and its high vulnerability to climate warming, microorganisms have been recognized as the key to mediate the impact of climate warming on permafrost region soil C [21]. In contrast to the previous observation at our study site that bacterial community taxonomic composition was unaltered by 1.5-year warming [4], we showed here that 5-year warming caused significant changes in the bacterial community composition, functional structure, and correlation networks (Table 1 and Additional file 1: Table S2). Our findings support the hypothesis that bacterial communities continue to evolve and diverge into new states (sensitivity) after long-term warming. Consequently, the higher functional capacity of microbial decomposition of soil C under warming contributes to higher soil respiration and CH_4_ flux, which in turn accelerates tundra C loss. Those observations are likely arising from changes by the winter warming treatment because soil temperature in the growing season remained unchanged (Additional file 1: Table S1).

Thaw of permafrost regions has long been considered to have profound effects on local hydrological, thermal, and C dynamics [3, 18, 22, 23]. We found that warming increased the thaw depth [24], which was the strongest factor linking to bacterial phylogenetic assembly (Fig. 4a), community composition (Additional file 1: Figure S6a), and network topology (Additional file 1: Figure S2). Consistently, deterministic processes (e.g., selection) played a more crucial role in shaping bacterial communities under warming (Additional file 1: Figure S5a). These results are consistent with a recent study of permafrost regions showing that changes in thaw depth induced changes in soil diazotrophic communities [25]. Moreover, the divergence of bacterial communities observed in this study under experimental warming manifested as increases of within-group β-diversity (Fig. 1c), might be a phenomenon generalizable to other ecosystems, since bacterial communities in a tallgrass prairie site were also divergent within warming replicates [26]. In sharp contrast, fungal communities remained unaltered by warming (Table 1). This could arise from the large variability of fungal communities as shown by the larger standard error of Faith’s *PD* for the fungi than for the bacteria (Fig. 1a, b).

The bacterial network of warmed samples exhibited higher average connectivity and shorter average geodesic distance than that of control samples (Additional file 1: Table S2), suggestive of a more complex network and denser interactions. The dense network is likely associated with deterministic processes (e.g., environmental filtering) [27]. Accordingly, we detected a higher contribution of deterministic processes under warming conditions (Additional file 1: Figure S5a).

Similar to the results of the 1.5-year warming at our study site [4], the relative abundance of functional genes associated with both aerobic and anaerobic C decomposition was increased by 5-year warming. These results could be crucial in assessing C dynamics in permafrost regions since the warming-induced thaw of permafrost regions exposes previously protected C stock to microbial activity. These findings also provide a mechanistic explanation for the recent observation that warming at our study site increased the annual cellulose decomposition rate at a soil depth of 0–10 cm by a factor of two [24]. In addition, the relative abundance of functional genes associated with recalcitrant C decomposition (e.g., aromatics and lignin, Fig. 2a) was increased by warming, which is in accordance with our finding that the relative abundance of the genus *Chitinophaga*, a strong chitinolytic taxa [28], was also increased by warming. Therefore, a potential increase in the decomposition of recalcitrant C is expected.

Field warming experiments have demonstrated that an initial increase of CO_2_ flux gradually subsides over time, returning to pre-warming values [29–34]. However, we observed persistent, enhanced ecosystem respiration after 5-year warming, which could result from a stimulated microbial decomposition of soil organic C (Additional file 1: Table S1). This phenomenon may arise from three mechanisms: (1) continuous warming increases the thaw depth, creating a crucial difference in the soil environment between warming and control plots, so acclimatization of microbial communities to warming is unlikely to occur; (2) since the temperature sensitivity of recalcitrant SOC is higher than labile SOC [16, 30], a higher microbial functional capacity of recalcitrant C decomposition under warming can aggravate soil C instability related to ecosystem respiration; and (3) the warming effect in permafrost regions is often more substantial for deeper soils [25], which contributes to ecosystem respiration. Therefore, we project that the soil microbial community would continue to provide positive feedback to climate warming.

All N cycling-associated genes exhibited higher relative abundance in warmed samples (Additional file 1: Figure S4a), which was consistent with the observations that both inorganic N availability and foliar N pools were increased by warming at our study site [12], and that soil nutrient contents were generally stimulated by warming in the tundra ecosystem [12, 13]. The larger nutrient pool available to plants could increase aboveground plant biomass (Additional file 1: Table S1). However, this higher plant productivity may only partially offset C loss, as a previous study of the Alaskan tundra observed a negative net ecosystem exchange due to a larger loss of C in deep soils than was increased by plant production [35]. Similarly, adding organic N to the active layer above the permafrost soils increased SOM decomposition by 2**–**3-fold [36]. Therefore, an increased soil nutrient availability associated with warming may further amplify C loss and consequently impose positive feedback to climate warming.

Collectively, our results show that 5-year warming significantly altered the bacterial composition and functional structure of microbial communities in permafrost regions, revealing an evolving sensitivity to warming. Soil thaw depth was the strongest factor shaping bacterial taxonomic composition, C decomposition potential, and network topological properties, demonstrating that warming-induced thaw of permafrost regions fundamentally restructures the associated bacterial communities. Therefore, we project that microbial responses to long-term warming will lead to positive feedback enhancing C decomposition in tundra regions.

## Methods

### Field site description and soil sampling

Established in 2008, the CiPEHR project is located within a discontinuous permafrost region in the northern foothills of the Alaska Range (~ 670 m elevation) at the Eight Mile study site, AK, USA (63°52′59′′ N, 149°13′32′′ W) [11, 37]. Soils in the experimental site are gelisols and comprise a 45–65-cm-thick organic horizon above a cryoturbated mineral mixture of glacial till and loess. The active layer, which thaws annually, is 50–60 cm thick. The site had a mean annual air temperature of − 1.45 ± 0.25 °C from 1977 to 2013 and a mean growing season precipitation of 216 ± 24 mm from 2004 to 2013. The dominant vegetation is a tussock-forming sedge, *Eriophorum vaginatum*. More detailed information on this site is available elsewhere [37].

Soils have been warmed since 2008 via snow fences (1.5 m tall, 8 m long), which act as insulators to increase the depth of the snow layer. Six snow fences are arranged in three blocks of two each, with each fence representing a warming-control plot pair. Each block is approximately 100 m apart and fences within a block are 5 m apart. Snow removal is conducted in the early spring (March 8–15) to avoid moisture and meltdown effects of the additional snow. In May 2013, surface soil samples at a depth of 0–15 cm were collected from both warming and control plots (6 replicates each), and then used for microbial community and environmental factor analyses.

### Measurement of environmental factors

Soil temperature at the depths of 5 and 10 cm was measured every half an hour in each plot using constantan-copper thermocouples and recorded using CR1000 data loggers (Campbell Scientific, Logan, UT, USA). Site-calibrated CS616 water content reflectometer probes (Campbell Scientific, Logan, UT, USA) were used to measure volumetric water content (moisture) at a depth of 0–15 cm. CS450 pressure transducers (Campbell Scientific, Logan, UT, USA) were used to continuously measure water table depth. The thaw depth was measured weekly during the growing season using a metal probe. Aboveground biomass was determined by a non-destructive point-frame method using a 60 × 60 cm frame with 8 × 8 cm grids, and species identity and tissue type (leaf, stem or fruit) for plants touching the rod (“hits”) were recorded as previously described [11]. Soil C and N contents were measured using an ECS 4010 Elemental Analyzer (Costech Analytical Technologies, Valencia, CA, USA). CH_4_ fluxes from each plot were measured as previously described [24], using a HP 5890 gas chromatograph (Hewlett-Packard, Palo Alto, CA, USA) equipped with a flame ionization detector and a molecular sieve 13X packed column. Ecosystem respiration was measured using an LI-820 infrared gas analyzer (LI-COR Biosciences, Lincoln, NE, USA) connected to a chamber placed on the plot base and covered by a dark tarp to exclude photosynthesis. The mean values of growing season soil temperature, soil moisture, water table depth, thaw depth, ecosystem respiration, and CH_4_ flux data from the 2012 growing season and winter soil temperature during the winter of late 2012–early 2013 were calculated.

### Soil DNA extraction

Soil DNA was extracted from 3 g of each soil sample by freeze-grinding mechanical cell lysis as described previously [38] and then purified with a PowerMax Soil DNA Isolation Kit (MO BIO, San Francisco, CA, USA). A NanoDrop ND-1000 spectrophotometer (NanoDrop Technologies Inc., Wilmington, DE, USA) was used to assess DNA quality using absorbance ratios of 260:280 and 260:230 nm. Final DNA concentrations were quantified using a Quant-iT PicoGreen dsDNA Assay kit (Invitrogen, Carlsbad, CA) with a FLUOstar OPTIMA fluorescence plate reader (BMG LabTech, Jena, Germany).

### High-throughput amplicon sequencing and raw data processing

The V4 hypervariable region of 16S rRNA gene was amplified with the primer pair 515F (5′-GTGCCAGCMGCCGCGGTAA-3′) and 806R (5′-GGACTACHVGGGTWTCTAAT-3′). The fungal internal transcribed spacer (ITS) was amplified with the primer pair ITS7F (5′-GTGARTCATCGARTCTTTG-3′) and ITS4R (5′-TCCTCCGCTTATTGATATGC-3′). A two-step PCR protocol was used to avoid bias introduced by long sequencing primers [39], which was an initial denaturation at 94 °C for 1 min, then 10 cycles (first step) or 20 cycles (second step) of 94 °C for 20 s, 53 °C (16S rRNA gene) or 52 °C (ITS) for 25 s, 68 °C for 45 s, followed by a final 10-min extension at 68 °C. The amplicons were paired-end sequenced (2 × 150) on a MiSeq sequencer (Illumina, San Diego, CA, USA). Sequences were denoised and processed on an online pipeline (www.ou.edu/ieg/tools/data-analysis-pipeline). Specifically, sequences were trimmed using BTRIM with a threshold quality score greater than 20 within a 5 bp window size and a minimum length of 100 bp. Forward and reverse reads with at least a 50 bp overlap and no more than 5% mismatches were joined using FLASH [40]. After removing sequences with ambiguous N bases, joined sequences with lengths between 245 and 260 bp for 16S rRNA, and between 100 and 450 bp for ITS were subjected to chimera removal by U-Chime as previously described [41, 42]. OTUs were clustered through Uclust at a 97% similarity level [41]. Taxonomic assignment was conducted through the RDP classifier [43] with a confidence cutoff of 0.5, and singletons were removed. The remaining sequences were randomly resampled to a depth of 34 673 reads per sample for 16S rRNA gene sequences, and 19 242 reads per sample for fungal ITS.

### GeoChip 5.0 analyses and raw data processing

Microbial functional genes were analyzed using the 180 K version of GeoChip 5.0M (Agilent Technologies Inc., Santa Clara, CA, USA), which contains 161 961 probes targeting 1 447 gene families involved in 12 major functional categories, such as C, N, P, and S cycling [19]. For each sample, 1 μg of soil DNA was labeled with Cy3 using random primers, dNTP solution and Klenow, purified with the Qiagen QIAquick Kit (Qiagen, Germantown, MD, USA) and dried using a SpeedVac (Thermo Fisher Scientific Inc., Waltham, MA, USA). Labeled samples were hybridized onto GeoChip at 67 °C in the presence of 10% formamide for 24 h. After hybridization, the arrays were washed, dried, and scanned at 100% laser power and photomultiplier tube on an MS200 Nimblegen microarray scanner (Roche Nimblegen, Madison, WI, USA). Scanned images were processed and transformed into signal intensities with Agilent’s Data Extraction software. Raw signal intensity files were uploaded onto an online pipeline (www.ou.edu/ieg/tools/data-analysis-pipeline) for further data quality filtering, normalization and data analyses. We normalized the signal intensity of each spot by relative abundance among all samples, removed spots with a signal-to-noise ratio (SNR) < 2, a signal intensity < 1.3 of background, or outliers based on judgements of 2 standard deviations.

### Molecular ecological network analysis

Phylogenetic molecular ecological networks (pMENs) were constructed from both the 16S rRNA gene and ITS sequences, using a random matrix theory (RMT)-based network pipeline (http://ieg4.rccc.ou.edu/MENA/) [44]. To ensure reliability, only OTUs detected in all six replicates were used for network construction. In brief, a matrix containing Spearman’s rho correlation between any pair of OTUs was generated. The threshold of similarity coefficients (*r* values of the Spearman’s rho correlation) for network construction was automatically determined when the nearest-neighbor spacing distribution of eigenvalues transitioned from Gaussian orthogonal ensemble to Poisson distributions [45]. Consequently, a threshold of 0.980 was used for bacterial networks of warming and control samples, 0.915 was used for the fungal network of control samples, and 0.920 was used for the fungal network of warming samples. To identify environmental factors important for network topology, environmental factors were also incorporated into networks, as RMT-based networks were designed to allow the use of multiple data types [45]. Random networks corresponding to all pMENs were constructed using the Maslov-Sneppen procedure with the same network size and average number of links to verify the system-specificity, sensitivity, and robustness of the empirical networks [46]. Network graphs were visualized with Cytoscape 3.5.1 software.

### Statistical analyses

Various statistical analyses were conducted with the package vegan (v2.3-2) [47] in R software version 3.2.2 [48]. Two-tailed Monte-Carlo permutation *t* tests and permutation analysis of variance (PERMANOVA) were used to examine the statistical significance of differences between microbial taxa, functional gene abundance or environmental factors (10000 permutations were generated for each test). Three complementary dissimilarity tests (multi-response permutation procedure [49], analysis of similarity [50], and non-parametric multivariate analysis of variance [51]) and detrended correspondence analysis [52] (DCA) were used to examine community differences. Canonical correspondence analysis (CCA) was used to detect linkages between microbial communities and environmental factors, with a threshold variance inflation factor of less than 20 to select independent environmental factors. To evaluate community assembly mechanisms, stochastic ratios were calculated with a modified stochastic ratio method [53] on the IEG Statistical Analysis Pipeline (www.ou.edu/ieg/tools/data-analysis-pipeline) based on phylogenetic (Beta-Mean Nearest Taxon Distance, βMNTD) metrics. Linear models were constructed to detect correlations among microbial communities and C fluxes with the package stats (v3.5.2) in R [48], and tested for significance by permutation tests with the package lmPerm (v2.1.0) [54].

## Supplementary information

**Additional file 1: Table S1.** Summary of environmental factors. **Table S2.** Major topological properties of the empirical pMENs of bacterial and fungal communities in the control and warming sites and the associated random networks. **Figure S1.** Microbial taxonomic composition of (a) bacterial communities and (b) fungal communities at the phylum level (for bacterial communities, *Proteobacterial* Classes are juxtaposed with other Phyla). Phyla with abundance less than 1% were combined to Others. **Figure S2.** Networks among environmental factors and microbial communities. (a) Bacterial communities from warming plots; (b) Bacterial communities from control plots; (c) fungal communities from warming plots, and (d) fungal communities from control plots. Red nodes represent environmental factors, blue nodes represent OTUs directly connected to environmental factors, grey nodes represent OTUs indirectly connected to environmental factors. Grey edges represent positive correlations, and red edges represent negative correlations. Abbreviations: Plant, aboveground plant biomass; Moisture, soil moisture; Bulk density, bulk soil density; C, soil total carbon; N, soil total nitrogen, and C/N, soil carbon/nitrogen ratio. **Figure S3.** The linear regressions between pairwise similarities of (a) bacterial community composition and functional structure (GeoChip data), and (b) fungal community composition and functional structure. Bray-Curtis distance was used for the similarity calculations. **Figure S4.** Differences of average normalized signal intensities of representative genes involved in (a) nitrogen cycling, (b) phosphorus cycling, and (c) sulfur cycling. (a) Red gene names represent genes with a higher average normalized signal intensity in warming samples, whose percentages of changes are indicated in parentheses. (b & c) Blue bars represent the average normalized signal intensity of gene probes in control samples, and red bars represent the average normalized signal intensity of gene probes in warming samples. Error bars represent standard errors. The differences between warming and control samples were tested using ANOVA, with * indicating *p* < 0.050. **Figure S5.** Overall community stochasticity on the basis of phylogenetic metric of (a) bacterial communities and (b) fungal communities. The data for each bar contains n = 15 within-group pairwise comparisons calculated from 6 biological replicates. **Figure S6.** Relationship between microbial community composition or functional structure and environmental factors revealed by canonical correspondence analysis (CCA) of (a) bacterial communities (red dots represent warming samples, and blue dots represent control samples) and environmental variables (arrows), (b) fungal communities and environmental variables; and (c) microbial functional structure and environmental variables. All CCA models are significant (*p* < 0.050).

## Data Availability

Raw sequences of 16S rRNA and ITS amplicon genes are available in NCBI SRA database (www.ncbi.nlm.nih.gov/sra) under accession number PRJNA506455. Raw data of GeoChip experiments can be accessed through the URL (129.15.40.254/NewIEGWebsiteFiles/publications/SupplData/CiPEHR_JiajieFENG_Raw_GeoChip_Data.txt) and (129.15.40.254/NewIEGWebsiteFiles/publications/SupplData/CiPEHR_JiajieFENG_Normalized_GeoChip_Data.txt).
