# Supplementary material for: Warming-induced permafrost thaw exacerbates tundra soil carbon decomposition mediated by microbial community
Source: Microbiome. 2020 Jan 17;8:3. doi: 10.1186/s40168-019-0778-3 (PMC6969446; doi:10.1186/s40168-019-0778-3)
Supplement: Supplementary file 2 — Additional file 1: Table S1. Summary of environmental factors. Table S2. Major topological properties of the empirical pMENs of bacterial and fungal communities in the control and warming sites and the associated random networks. Figure S1. Microbial taxonomic composition of (a) bacterial communities and (b) fungal communities at the phylum level (for bacterial communities, Proteobacterial Classes are juxtaposed with other Phyla). Phyla with abundance less than 1% were combined to Others. Figure S2. Networks among environmental factors and microbial communities. (a) Bacterial communities from warming plots; (b) Bacterial communities from control plots; (c) fungal communities from warming plots, and (d) fungal communities from control plots. Red nodes represent environmental factors, blue nodes represent OTUs directly connected to environmental factors, grey nodes represent OTUs indirectly connected to environmental factors. Grey edges represent positive correlations, and red edges represent negative correlations. Abbreviations: Plant, aboveground plant biomass; Moisture, soil moisture; Bulk density, bulk soil density; C, soil total carbon; N, soil total nitrogen, and C/N, soil carbon/nitrogen ratio. Figure S3. The linear regressions between pairwise similarities of (a) bacterial community composition and functional structure (GeoChip data), and (b) fungal community composition and functional structure. Bray-Curtis distance was used for the similarity calculations. Figure S4. Differences of average normalized signal intensities of representative genes involved in (a) nitrogen cycling, (b) phosphorus cycling, and (c) sulfur cycling. (a) Red gene names represent genes with a higher average normalized signal intensity in warming samples, whose percentages of changes are indicated in parentheses. (b & c) Blue bars represent the average normalized signal intensity of gene probes in control samples, and red bars represent the average normalized signal intensity of gene pro [file 40168_2019_778_MOESM2_ESM.docx]

**Table S1.** Summary of environmental factors.

| Environmental factors^a^ | Control | Warming |
| --- | --- | --- |
| Winter soil temperature (°C) | **-1.95 ± 0.25** | **-1.32 ± 0.13** |
| Growing season soil temperature (°C) | 6.15 ± 0.46 | 6.20 ± 0.33 |
| Soil moisture (%) | 37.77 ± 1.39 | 42.54 ± 2.90 |
| Water table depth (cm) | 23.82 ± 1.19 | 21.79 ± 1.90 |
| Thaw depth (cm) | **40.98 ± 0.70** | **52.35 ± 2.65** |
| Aboveground plant biomass (g/m^2^) | **1619.0 ± 69.2** | **2025.0 ± 179.5** |
| Soil carbon/nitrogen ratio | 30.03 ± 2.52 | 30.41 ± 2.08 |
| Ecosystem respiration (mg (CO_2_-C) m^-2^ h^-1^) | **94.74 ± 6.02** | **163.68 ± 9.85** |
| Soil CH_4_ flux (µg C m^-2^ h^-1^) | **96.97 ± 32.28** | **309.10 ± 49.62** |

^a^Mean value and standard error of n = 6 biological replicates are shown, and for Soil CH_4_ flux there are n = 6 biological replicates of control and n = 4 of warming plots, with one missing data point (warming biological replicate 1) and one data point identified as an outlier by boxplot (1871.58 µg C m^-2^ h^-1^, warming biological replicate 2) excluded. Differences between warming and control plots were examined by two-tailed permutation *t-*tests. Bold values indicate *p* < 0.050.

**Table S2**. Major topological properties of the empirical pMENs of bacterial and fungal communities in the control and warming sites and the associated random networks.

| Community | Empirical networks | | | | |  | | | |  | Random networks^a^ | | |
| --- | --- | --- | --- | --- | --- | --- | --- | --- | --- | --- | --- | --- | --- |
|  | No. of original OTUs | Similarity threshold | Network size | R^2^ of power law | Average connectivity | | Average geodesic distance | Average clustering coefficient | Modularity(No. of modules) |  | Average geodesic distance ± SD | Average clustering coefficient ± SD | Average modularity± SD |
| Control-bacteria | 1877 | 0.980 | 1644 | 0.742 | 3.526^b^ | | 5.202^b,c^ | 0.476^c^ | 0.954^c^(222) |  | 5.366±0.081 | 0.003±0.001 | 0.578±0.003 |
| Warming-bacteria | 1824 | 0.980 | 1568 | 0.819 | 4.305^b^ | | 4.885^b,c^ | 0.486^c^ | 0.926^c^(189) |  | 4.658±0.060 | 0.005±0.001 | 0.494±0.003 |
| Control-fungi | 118 | 0.915 | 84 | 0.870 | 3.119^b^ | | 3.384^b,c^ | 0.304^c^ | 0.718^c^(17) |  | 3.530±0.115 | 0.049±0.015 | 0.511±0.014 |
| Warming-fungi | 107 | 0.920 | 88 | 0.886 | 1.909^b^ | | 1.709^b,c^ | 0.249^c^ | 0.921^c^(21) |  | 6.161±0.822 | 0.014±0.010 | 0.778±0.016 |

^a^The random networks were generated by rewiring all the links of a pMEN with an identical number of nodes and links to the corresponding empirical pMEN.

^b^Significant difference (*p* < 0.050) between control and warming networks.

^c^Significant difference (*p* < 0.050) between empirical networks and random networks.


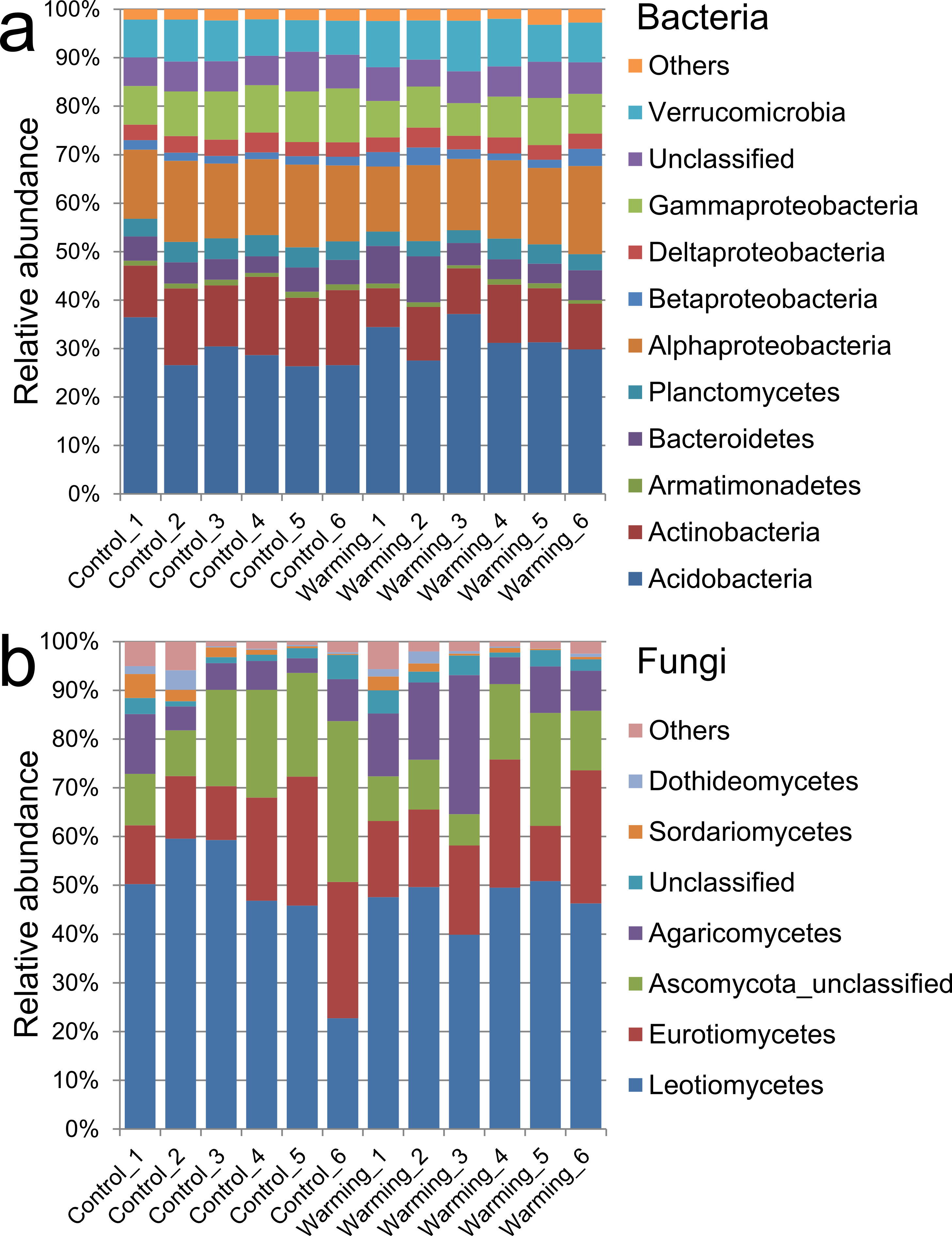


**Figure S1**. Microbial taxonomic composition of (a) bacterial communities and (b) fungal communities at the phylum level (for bacterial communities, *Proteobacterial* Classes are juxtaposed with other Phyla). Phyla with abundance less than 1% were combined to Others.

**
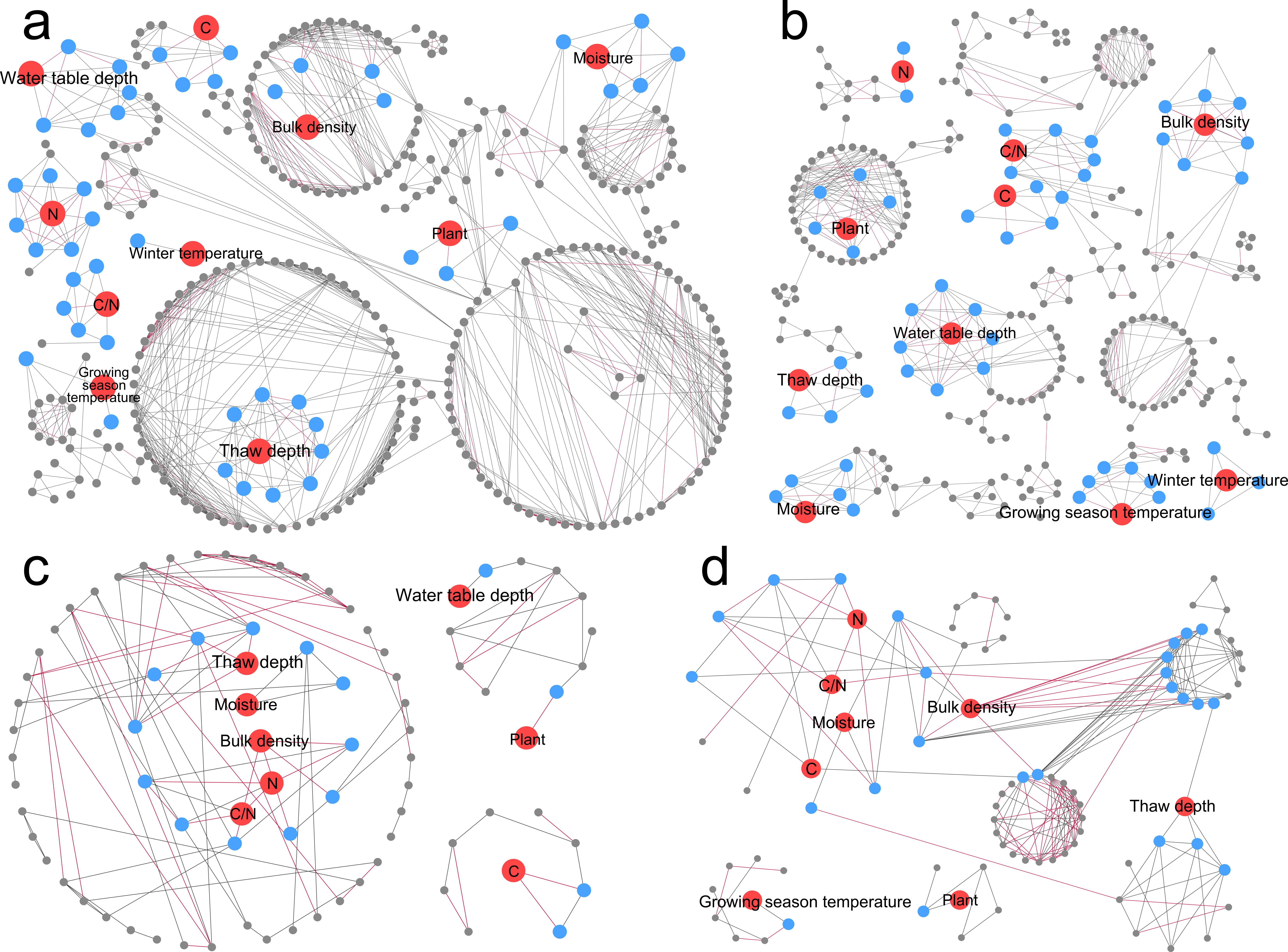
**

**Figure S2**. Networks among environmental factors and microbial communities. (a) Bacterial communities from warming plots; (b) Bacterial communities from control plots; (c) fungal communities from warming plots, and (d) fungal communities from control plots. Red nodes represent environmental factors, blue nodes represent OTUs directly connected to environmental factors, grey nodes represent OTUs indirectly connected to environmental factors. Grey edges represent positive correlations, and red edges represent negative correlations. Abbreviations: Plant, aboveground plant biomass; Moisture, soil moisture; Bulk density, bulk soil density; C, soil total carbon; N, soil total nitrogen, and C/N, soil carbon/nitrogen ratio.


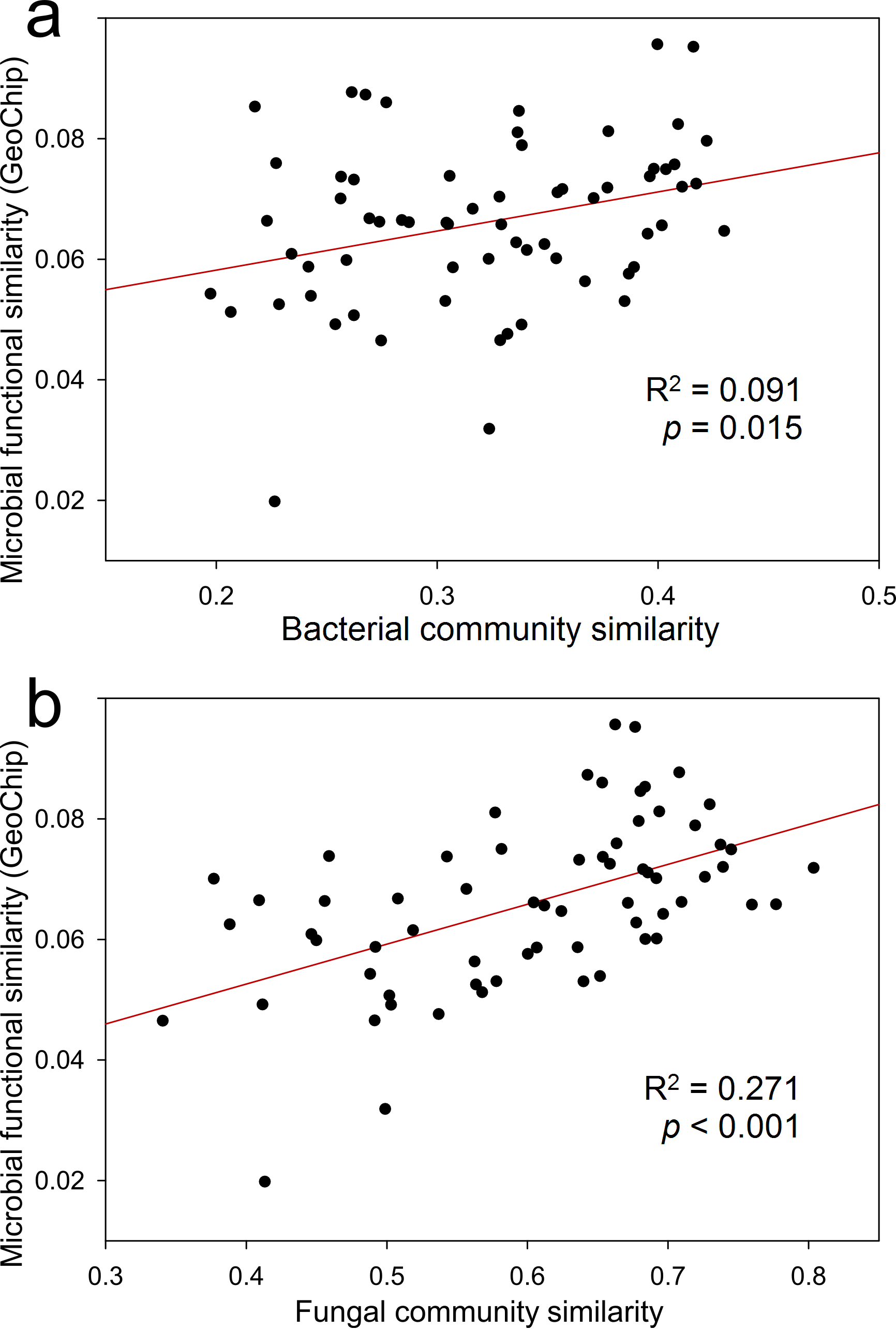


**Figure S3**. The linear regressions between pairwise similarities of (a) bacterial community composition and functional structure (GeoChip data), and (b) fungal community composition and functional structure. Bray-Curtis distance was used for the similarity calculations.


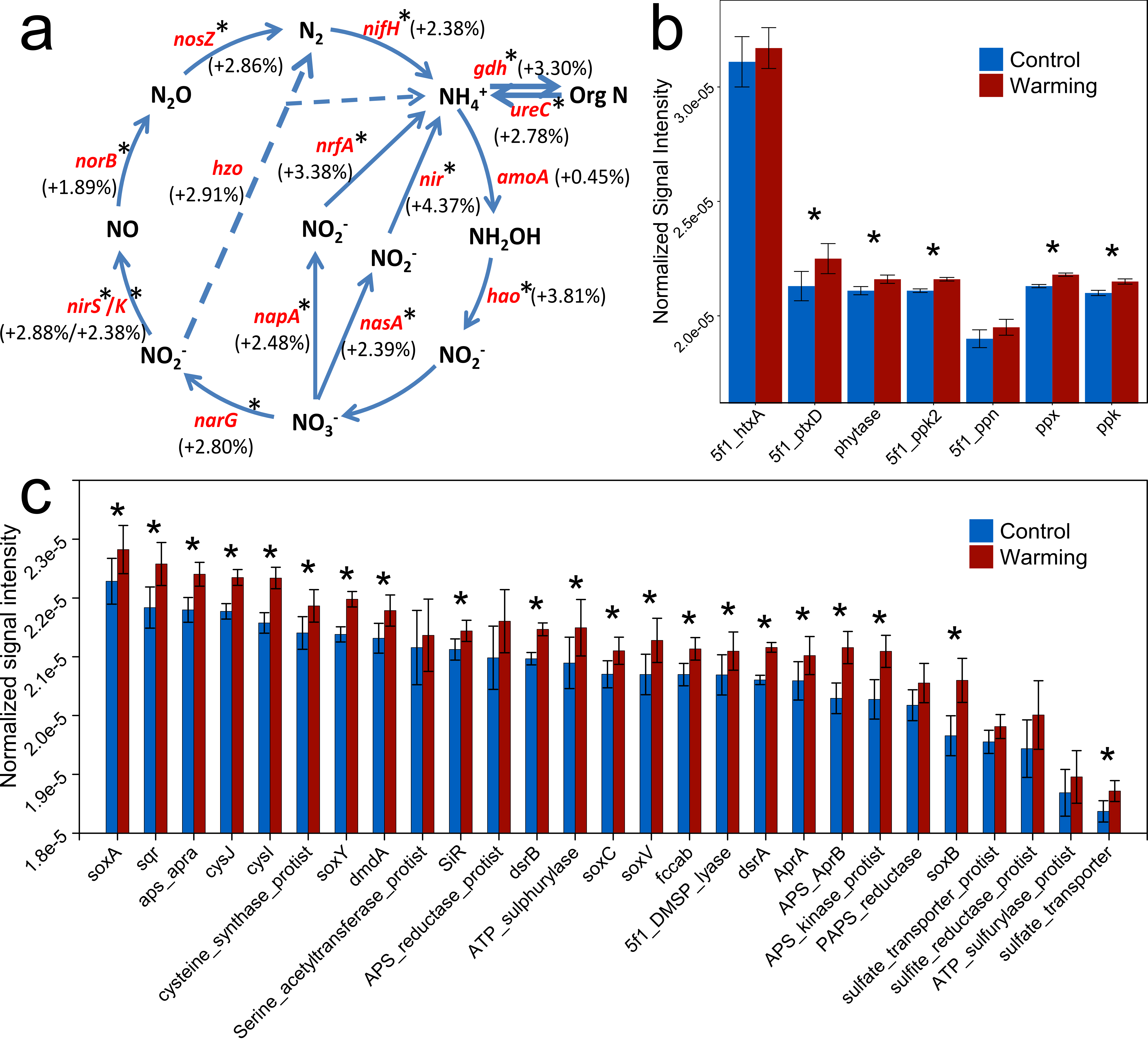


**Figure S4**. Differences of average normalized signal intensities of representative genes involved in (a) nitrogen cycling, (b) phosphorus cycling, and (c) sulfur cycling. (a) Red gene names represent genes with a higher average normalized signal intensity in warming samples, whose percentages of changes are indicated in parentheses. (b & c) Blue bars represent the average normalized signal intensity of gene probes in control samples, and red bars represent the average normalized signal intensity of gene probes in warming samples. Error bars represent standard errors. The differences between warming and control samples were tested using ANOVA, with * indicating *p* < 0.050.


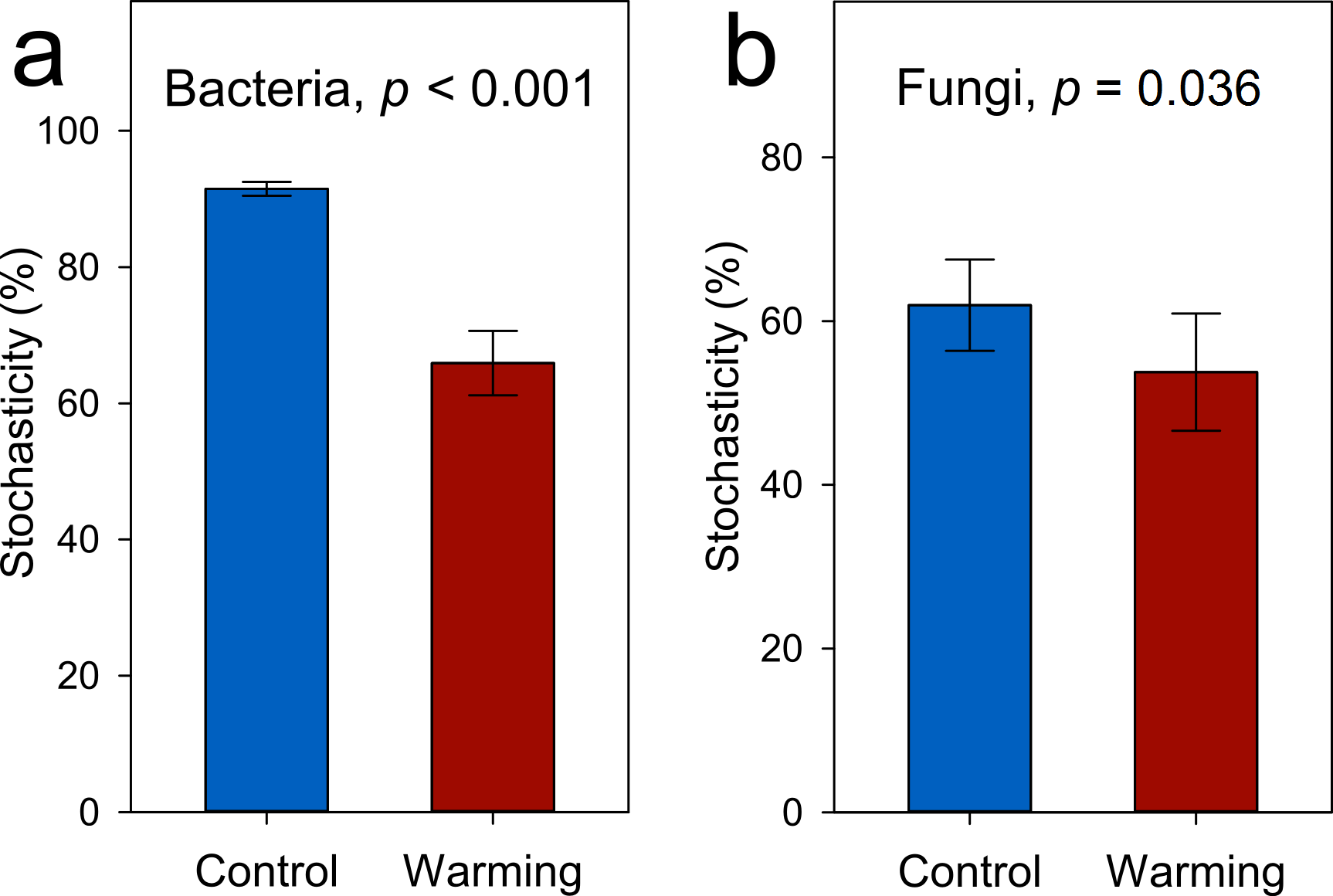


**Figure S5.** Overall community stochasticity on the basis of phylogenetic metric of (a) bacterial communities and (b) fungal communities. The data for each bar contains n = 15 within-group pairwise comparisons calculated from 6 biological replicates.


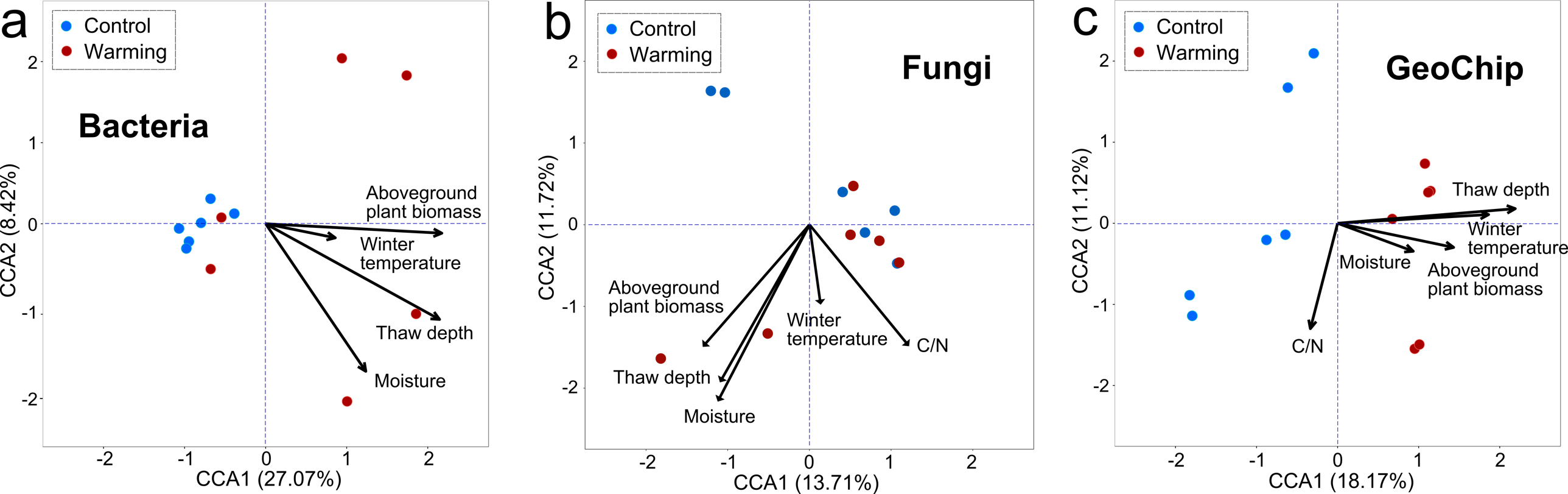


**Figure S6**. Relationship between microbial community composition or functional structure and environmental factors revealed by canonical correspondence analysis (CCA) of (a) bacterial communities (red dots represent warming samples, and blue dots represent control samples) and environmental variables (arrows), (b) fungal communities and environmental variables; and (c) microbial functional structure and environmental variables. All CCA models are significant (*p* < 0.050).
